# The Gut Microbiota Activates AhR Through the Tryptophan Metabolite Kyn to Mediate Renal Cell Carcinoma Metastasis

**DOI:** 10.3389/fnut.2021.712327

**Published:** 2021-08-11

**Authors:** Guoyu Dai, Xiang Chen, Yao He

**Affiliations:** Department of Urology, Xiangya Hospital, Central South University, Changsha, China

**Keywords:** rcc, AhR, KYN, EMT, gut microbiota

## Abstract

**Background:** The incidence of renal cell carcinoma (RCC) is increasing year by year. It is difficult to have complete treatment so far. Studies have shown that tryptophan metabolite Kynurenine (Kyn) affects cell proliferation, migration, apoptosis, adhesion, and differentiation. Our aim is to explore whether Kyn activates aromatic hydrocarbon receptor (AhR) to mediate RCC metastasis.

**Methods:** We collected RCC tissues and feces from RCC patients. 16S rRNA technology was performed to analyze the gut microbial composition of RCC patients. LC-MS/MS was used to analyze the gut microbial metabolites. The AhR was inhibited and treated with Kyn. Immunofluorescence was used to measure the degree of AhR activation. The migration and invasion ability of 786-O cells was tested by Transwell assay. Flow cytometry and cell cycle assay were utilized to observe the apoptosis and cycle of 786-O cells. CCK-8 assay was used to detect 786-O cells proliferation. qRT-PCR and Western blot were used to detect AhR and EMT-related genes expression level.

**Results:** AhR expression was up-regulated in RCC tissues. RCC gut microbiota was disordered. The proportion of Kyn was increased in RCC. After being treated with Kyn, the migration, invasion, and proliferation ability of 786-O cells were decreased. Furthermore, the expression of EMT-related protein E-cadherin decreased, and the expression of N-cadherin and Vimentin increased. The proportion of 786-O cells in the S phase increased. The apoptosis rate of 786-O cells was inhibited.

**Conclusion:** The tryptophan metabolite Kyn could activate AhR. Kyn could promote 786-O cells migration and invasion. Gut microbiota could activate AhR through its tryptophan metabolite Kyn to mediate RCC metastasis.

## Introduction

Renal cell carcinoma (RCC) is still an elusive cancer in lack of biomarkers. It was the eighth most common malignant tumor in the United States (1). In addition to the increase in newly diagnosed cases, RCC patients' prevalence and overall survival rate have also increased significantly (2). Studies have shown a link between the gut microbiota and metastatic RCC (mRCC). Evidence showed that RCC patients have a lower abundance of *bifidobacteria*, compared with healthy adults (3). However, it needs experimental verification about whether there is a connection between the gut microbiota and RCC needs to.

Many life activities are mediated by metabolites of gut microbiota. Tryptophan is an essential aromatic amino acid, and it is considered necessary in many metabolites between gut microbiota and the host (4, 5). Many tryptophan metabolites derived from abundant microbiota exhibit the activation potential of aromatic hydrocarbon receptor (AhR) (6). Some endogenous tryptophan metabolites are recognized as AhR ligands, including tryptamine (TRA), indole, 5-hydroxyindole-3-acetic acid (5-HIAA), Kynurenine (Kyn), kynurenic acid (KA), and xanthine acid (XA) (7, 8). Tryptophan metabolites as ligands can activate AhR signals in many diseases, such as inflammation, oxidative stress damage, cancer, aging-related diseases, cardiovascular disease (CVD), and chronic kidney disease (CKD) (4). We screened tryptophan metabolites to verify the regulatory relationship between tryptophan metabolites and AhR.

AhR is a cytoplasmic ligand-activated transcription factor involved in various cellular processes. It can mediate the toxicity (including carcinogenicity) of polycyclic aromatic hydrocarbons and induce many enzymes expression. It can participate in critical biological processes, such as signal transduction, cell differentiation, and cell apoptosis (9). Recent studies have shown that AhR is related to CVD, CKD, and RCC (10). Tryptophan catabolites can activate AhR to enhance tumor malignancy and inhibit anti-tumor immunity (11, 12). More studies revealed AhR can be activated by many endogenous ligands. Different ligands bind and activate AhR, which can translocate AhR to the nucleus and induce a series of genes expression (8).

Epithelial-mesenchymal transition (EMT) is a process in which epithelial cells lose their polarized structure and gain the migration and invasion ability. It is believed to be the cause of cancer metastasis (13). EMT biomarkers such as Vimentin, N-cadherin, and MMP9 are overexpressed in cancer and are involved in promoting cancer cells metastasis (14). Many studies have shown that AhR activity leads to loss of cell contact inhibition and changes in extracellular matrix remodeling (15). This study intends to explore the internal relationship between metastatic RCC and the gut microbiota and its metabolism (tryptophan metabolism) and verify whether the tryptophan metabolite Kyn promotes EMT and RCC pathological process by activating AhR.

## Results

### High Expression of AhR in RCC Tissues

**Figure 1:**
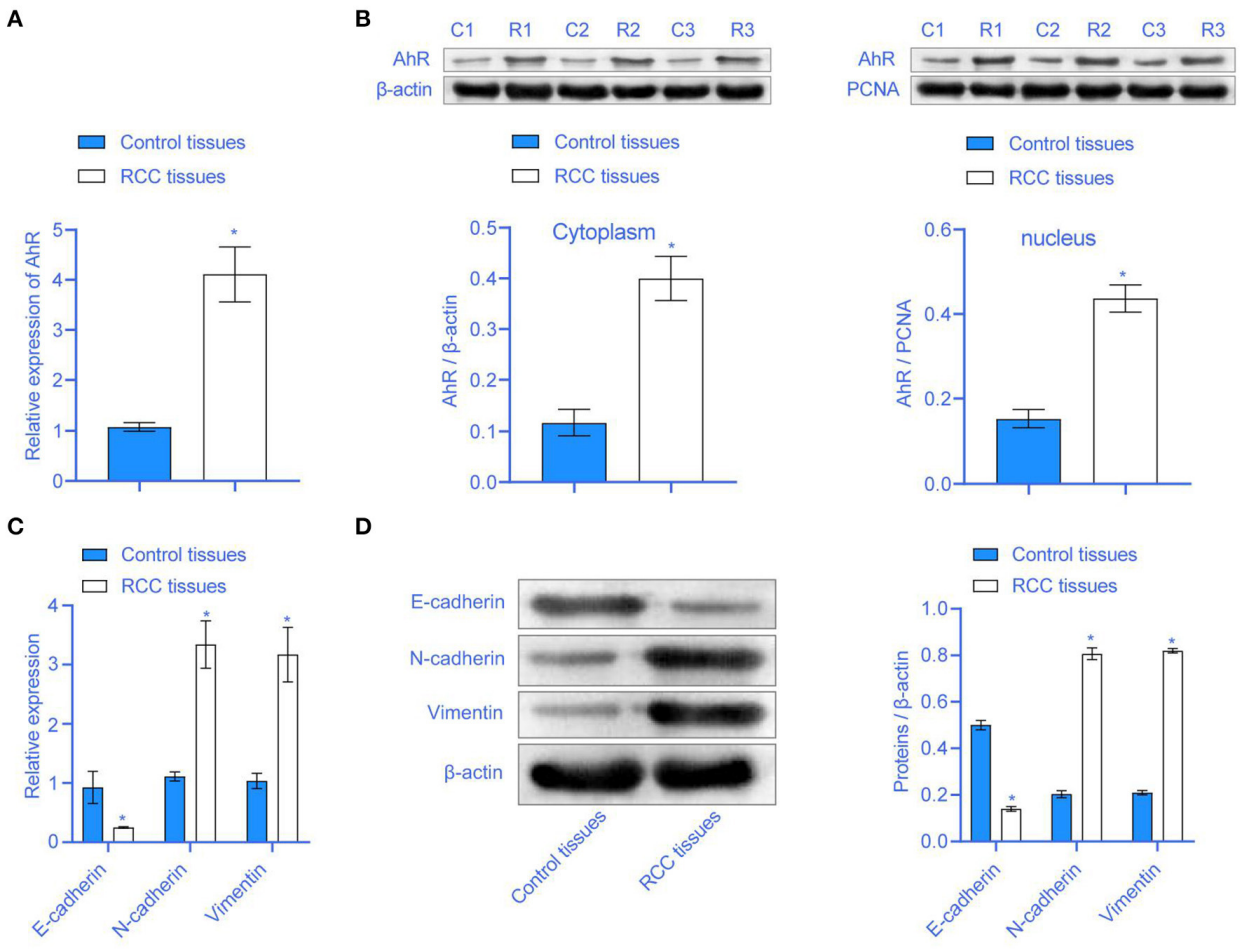
The expression of AhR was higher in RCC. **(A)** The mRNA expression of AhR was higher in the RCC tissues group. **(B)** The expression of AhR protein in the cytoplasm and nucleus. **(C,D)** E-cadherin, N-cadherin, and Vimentin were abnormally expressed in RCC tissues group * compared with Control tissues group, *P* < 0.05.

The clinical characteristics of all subjects were presented in Table 1. To study whether AhR expression in RCC was abnormal, we used qRT-PCR and Western blot to detect AhR expression. Compared with the Control tissues group, the AhR mRNA expression in the RCC tissues group was significantly increased (Figure 1A). AhR protein expression was increased in both the cytoplasm and the nucleus (Figure 1B). It showed that AhR expression was abnormally increased in RCC. RCC was often accompanied by EMT conversion (16). We next detected the expression of E-cadherin, N-cadherin, and Vimentin related to EMT. Compared with the Control tissues group, E-cadherin expression in the RCC tissues group was inhibited, but N-cadherin and Vimentin expressions were significantly up-regulated (Figures 1C,D). It showed that EMT accompanied the RCC patients, and AhR expression was abnormal.

**Table 1:** Characteristics of patients with RCC.

**Characteristics**	**Total (*n* = 10)**
**Sex**, ***n***	
Men	6
Women	4
**Age at enrollment, years**, ***n***	
<60	8
≥60	2
**Smoking history**, ***n***	
Yes	4
No	6
**Cancer stage**, ***n***	
T1N0M0	10
Other	0

### The Diversity of Gut Microbiota in RCC Changed

**Figure 2:**
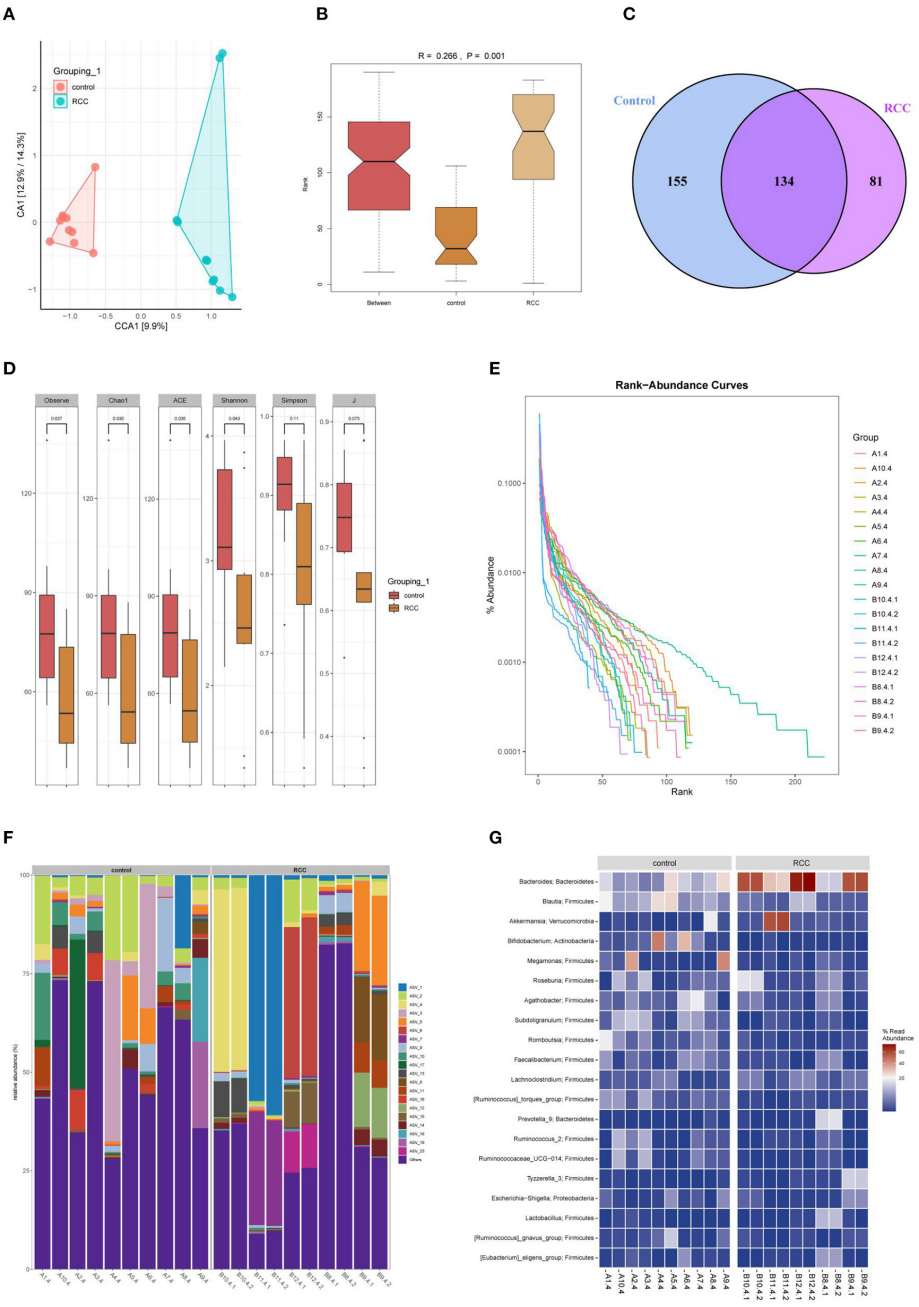
Gut microbiota imbalance caused by RCC. **(A)** The similarity between the Control tissues and RCC tissues groups. **(B)** The difference between the two groups. **(C)** OUT Venn diagram. **(D)** Box chart. **(E)** Rank-abundance curve. **(F)** Histogram of the distribution of phylum species. **(G)** Heat map of phylum-species distribution.

Next, we aim to explore whether RCC affected the gut microbiota of RCC patients. PCA analysis showed that the microbial community similarity of the clinical samples of the Control tissues group and the RCC tissues group was low (Figure 2A). Anosim analysis further helped obtain an R-value of 0.266, showing that the difference between groups was more significant than the difference within groups (Figure 2B). The OUT Venn diagram showed that the Control tissues group had 155 unique OUT numbers, the RCC tissues group had 819 unique OUT numbers, and the two groups had 134 OUT numbers in total (Figure 2C). Chao1, Shannon, and Simpson's indexes showed the difference between the two groups was significant (Figure 2D) (*P* < 0.05). The Rank-abundance curve indicated that the RCC tissues group curve had a larger range on the horizontal axis, and the species richness was higher (Figure 2E). The abundance bar graph showed a significant difference in species composition between the Control tissues group and RCC tissue group (Figure 2F). The heat map showed that compared with the Control tissues group, the abundance of *Bacteroides* and *Akkermansia* was increased significantly in RCC tissues group, while the abundance of *Blautia, Bifidobacterium*, and *Megamonas* was decreased significantly (Figure 2G). As shown above, the gut microbiota of RCC patients was imbalanced.

### Tryptophan Metabolites From Gut Microbiota Was Related to RCC

**Figure 3:**
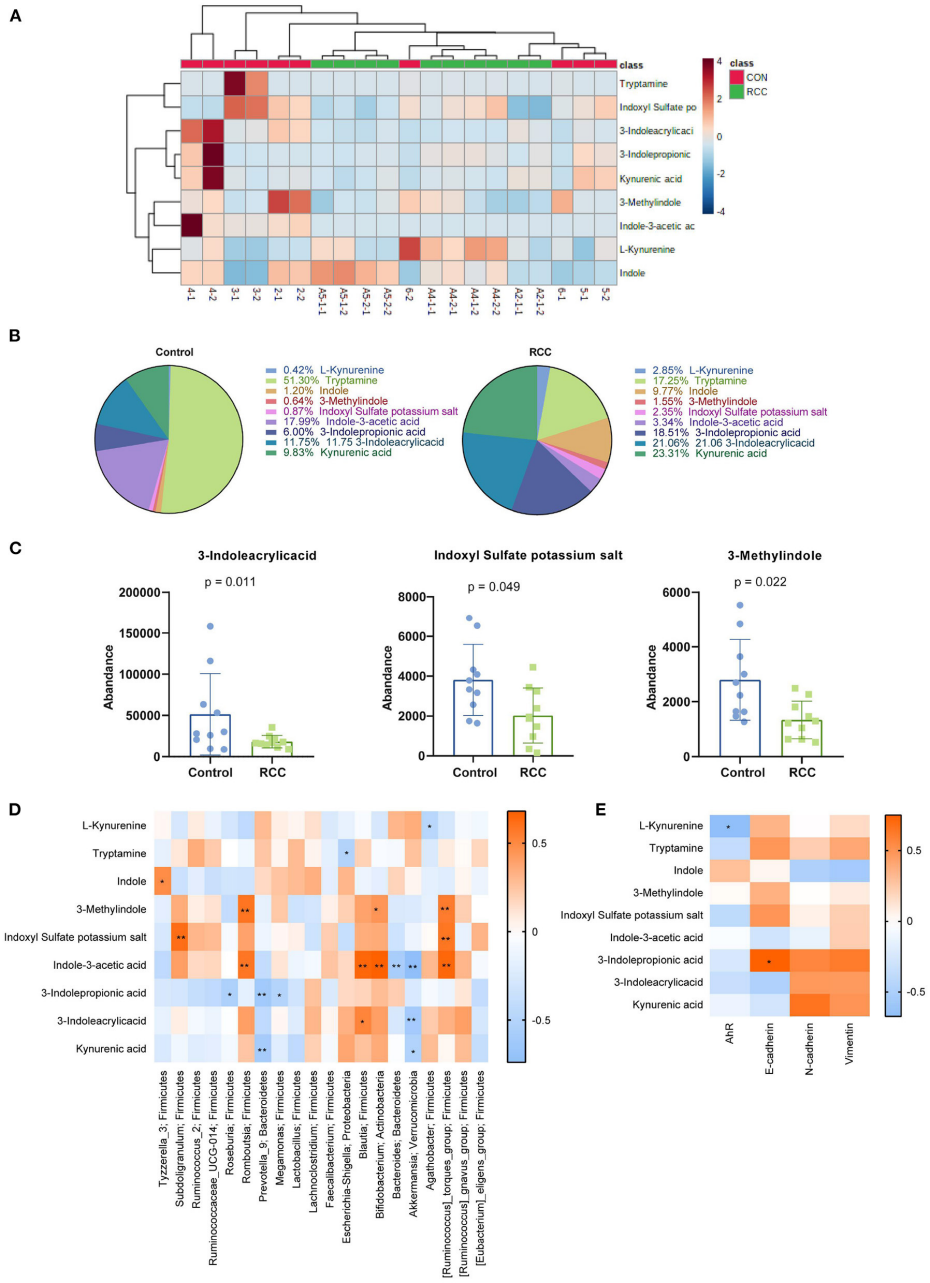
The content of Kyn was high in RCC. **(A)** Heat map of metabolites (2-1, 2-2, 3-1, 3-2, 4-1, 4-2.5-1, 5-2, 6-1, and 6-2 belong to control group, A2-1-1, A2-1-2, A4-1-1, A4-1-2, A4-2-1, A4-2-2, A5-1-1, A5-1-2, A5-2-1, and A5-2-2 belong to RCC group). **(B)** Percentage of nine tryptophan metabolites. **(C)** Three metabolites with reduced content in RCC. **(D)** The relationship between 9 tryptophan metabolites and the top 20 gut microbiota. **(E)** The correlation between the tryptophan metabolites and AhR, E-cadherin, N-cadherin, and Vimentin.

The above experiments indicated that RCC could lead to gut microbiota disturbance. We detected tryptophan metabolites. First, we uploaded the metabolic data on the online website (https://www.metaboanalyst.ca/MetaboAnalyst/ModuleView.xhtml) to get the heat map (Figure 3A). The heat map showed our nine tryptophan metabolites (L-Kynurenine, Tryptamine, Indole, 3-Methylindole, Indoxyl Sulfate potassium, salt, Indole-3-acetic acid, 3-Indolepropionic acid, and 3-Indoleacrylicacid Kynurenic acid) in different groups were different. Percentage of the distribution ratio of tryptophan metabolites in the Control tissues group and the RCC tissues group indicated meaningful differences (Figure 3B). Then, original data were concluded. 3-Indoleacrylicacid, Indoxyl Sulfate potassium salt, and 3-Methylindole were significantly reduced (Figure 3C). The Spearman's rank correlation was performed to analyze the correlation between the top 20 gut microbiota and nine tryptophan metabolites. The results showed that L-Kynurenine was negatively correlated with *Agathobacter*. Tryptamine was negatively correlated with *Escherichia-Shigella*. Indole was positively correlated with *Tyzzerella_3*. 3-Methylindole was positively correlated with *Romboutsia, Bifidobacterium*, and *[Ruminococcus]_torques_group*. Indoxyl Sulfate potassium salt was positively correlated with Subdoligranulum and *[Ruminococcus]_torques_groups*. Indole-3-acetic acid was positively correlated with *Romboutsia, Blautia, Bifidobacterium*, and *[Ruminococcus]_torques_group*. Indole-3-acetic acid was negatively correlated with *Bacteroides* and *Akkermansia*. 3-Indolepropionic acid was negatively correlated with *Roseburia, Prevotella_9, and Megamonas*. 3-Indoleacrylicacid was positively correlated with *Blautia*. 3-Indoleacrylicacid was negatively correlated with *Akkermansia*. *Kynurenic acid* was negatively correlated with *Prevotella_9* and *Akkermansia*. The above results indicated that gut microbiota imbalance in RCC patients might lead to tryptophan metabolites disorders (Figure 3D). Next, we analyzed the correlation between the tryptophan metabolites and AhR, E-cadherin, N-cadherin, and Vimentin. The results revealed AhR was significantly negatively correlated with L-Kynurenine. E-cadherin was significantly positively correlated with 3-Indolepropionic acid (Figure 3E). The above indicated that the disturbance of tryptophan metabolites from gut microbiota was related to the abnormal expression of EMT and AhR in RCC.

### Kyn Could Activate AhR to Inhibit 786-O Cells Apoptosis

**Figure 4:**
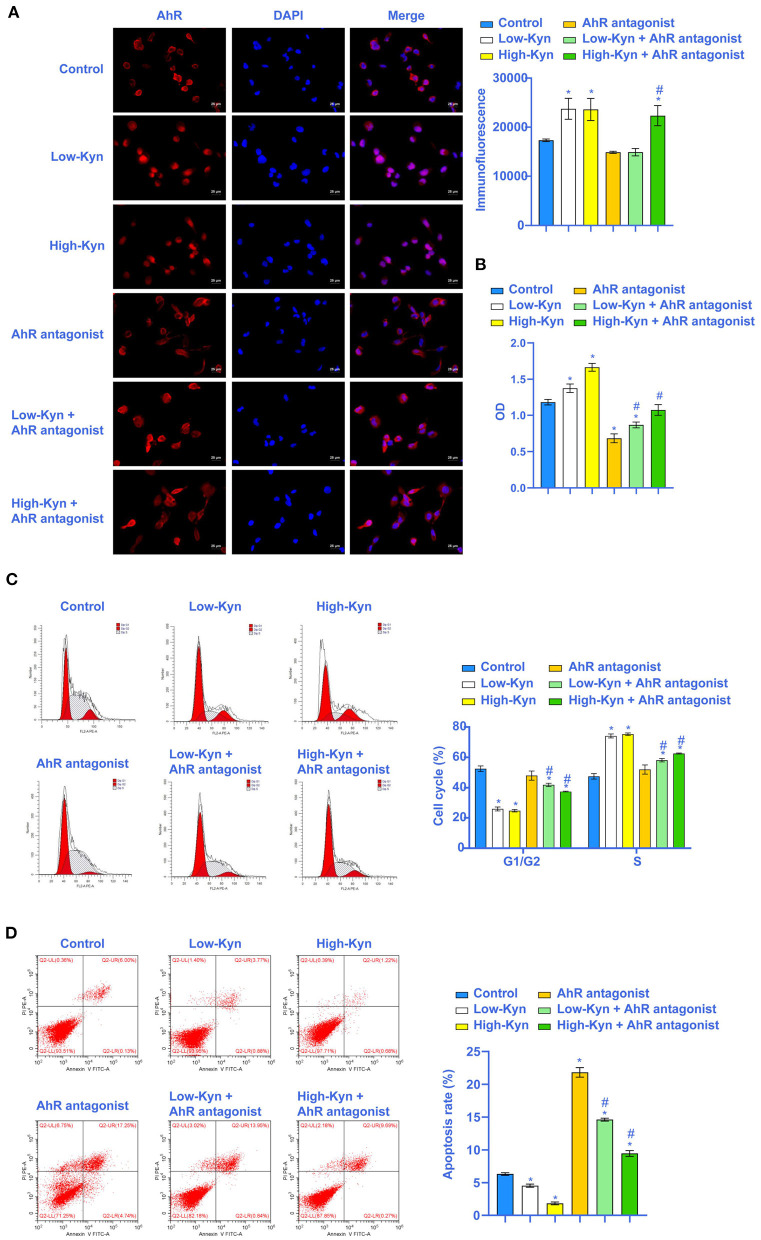
Kyn could inhibit 786-O cells apoptosis. **(A)** AhR was activated by Kyn. **(B)** CCK-8 assay was used to measure the cell vitality. **(C)** Kyn could stabilize the 786-O cells cycle (×200, Scale bar = 50μm). **(D)** Flow cytometry was used to measure the apoptosis rate of 786-O cells. * Compared with the Control group, *P* < 0.05. # Compared with the AhR antagonist group, *P* < 0.05.

To further explore the effect of tryptophan metabolites on RCC, we inhibited AhR. We treated 786-O cells with different concentrations of Kyn. The immunofluorescence results showed that Kyn could activate AhR in 786-O cells (Figure 4A). Cell viability assay showed that 786-O cells viability was increased in Low-Kyn and High-Kyn groups compared with the Control group. Compared with the AhR antagonist group, the 786-O cells viability in the Low-Kyn + AhR antagonist and High-Kyn + AhR antagonist groups was increased (Figure 4B). Then, we checked the cell cycle and cell apoptosis. Kyn was added to treat 786-O cells, and the results suggested that the cells number arrested in the G1/G2 phase decreased, and the cells number in the S phase increased. The AhR of 786-O cells was inhibited. Then Kyn was added to treat 786-O cells. The cells number arrested in the G1/G2 phase was also significantly reduced, and cells number in the S phase was increased substantially (Figure 4C). It indicated that Kyn could regulate the normal life cycle of 786-O cells. Flow cytometry was used to measure the 786-O cells apoptosis (Figure 4D). The results suggested that Kyn could effectively inhibit 786-O cells apoptosis. In summary, Kyn could activate AhR to inhibit 786-O cells apoptosis.

### Kyn Could Promote 786-O Cells Migration and Invasion by Activating AhR

**Figure 5:**
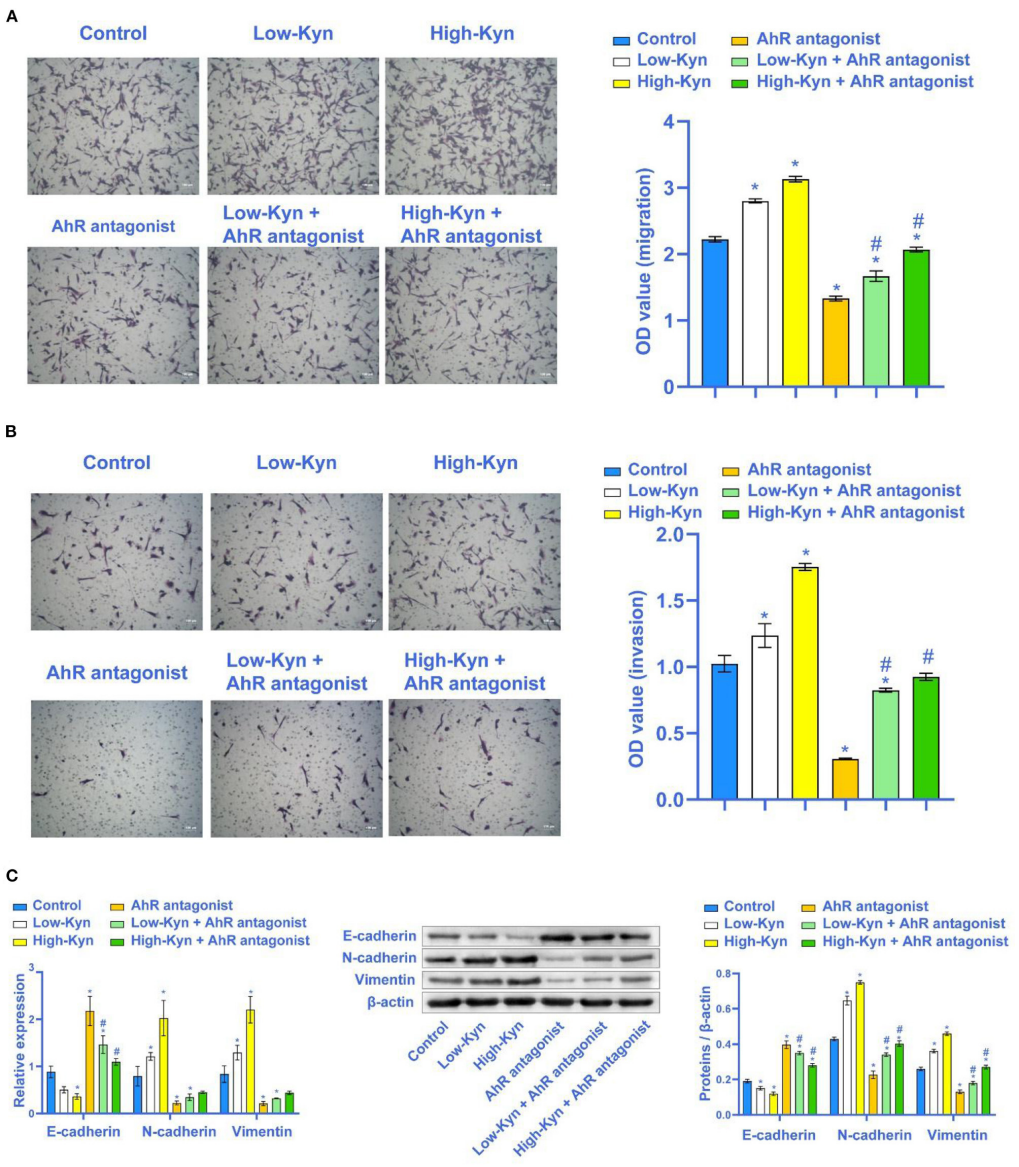
Kyn could promote the migration and invasion of 786-O cells. **(A)** and **(B)** Transwell was used to detect the migration and invasion ability of 786-O cells (×100, Scale bar = 100μm). **(C)** E-cadherin was inhibited by Kyn. N-cadherin and Vimentin were activated by Kyn. * Compared with the Control group, *P* < 0.05. # Compared with the AhR antagonist group, *P* < 0.05.

The above experimental results indicated that Kyn could inhibit the 786-O cells apoptosis. Next, we tested whether Kyn could affect the invasion and EMT process of 786-O cells. Compared with the Control group, 786-O cells migration and invasion ability in the Low-Kyn and High-Kyn groups was increased. Compared with the AhR antagonist group, 786-O cells migration and invasion ability in the Low-Kyn + AhR antagonist and High-Kyn + AhR antagonist groups were increased. It suggested that Kyn could promote the 786-O cells migration and invasion (Figures 5A,B). Finally, qRT-PCR and Western blot were used to detect the expression of E-cadherin, N-cadherin, and Vimentin related to EMT genes. Kyn was added to treat 786-O cells. The E-cadherin expression in 786-O cells was inhibited, but N-cadherin and Vimentin expressions were significantly up-regulated (Figure 5C). In short, Kyn could activate AhR to promote the migration, invasion, and EMT process of 786-O cells.

## Materials and Methods

### Clinical Sample Collection

We collected 10 RCC tissues samples and Control tissues (at least 5 cm from the tumor tissues) from the Xiangya hospital central south university. We also collected patient feces. These samples came from RCC patients after surgery. None of these patients received chemotherapy or radiotherapy before surgery. Patients with infectious diseases, autoimmune diseases, or multiple primary cancers were excluded. We also collected 10 normal human feces. Before the sample collection, all subjects did not receive antibiotics or similar drugs. The research was approved by the Ethics Association and related hospitals.

### Cell Culture

Human RCC 786-O cells were cultured in a T25 cell culture flask (690175, Greiner Bio-One Vilvoorde, Belgium) supplemented with 100 U/ml penicillin. The cells were cultured in a 5% CO_2_ medium containing 10% fetal bovine serum (FBS) (Gibco). The medium was replaced with a serum-free medium. 786-O cells were inoculated into a six-well culture plate and incubated for at least 12. The cells were grouped as the Control tissues group (786-O cells), the Low-Kyn group (786-O cells were cultured in 0.2 mmol/L Kyn for 24 h), the High-Kyn group (786-O cells were cultured in 2 mmol/L Kyn for 24 h), the AhR antagonist group (786-O cells were pretreated with DMF (3′, 4′-dimethoxyflavone) for 12 h), the Low-Kyn + AhR antagonist group (786-O cells were pretreated with DMF and cultured at 0.2 mmol/L Kyn), and the High-Kyn + AhR antagonist group (786-O cells were pretreated with DMF and cultured under two mmol/L Kyn) (17).

### Bacterial 16S rRNA Data Processing and Analysis

We de-joined and filtered the raw data with low-quality. The representative sequence of each out was annotated. We have separated and filtered the low-quality raw data. We have annotated the representative sequence of each OTU. The random sampling method was adopted, and the OTU analysis was performed with the number of effective sequences drawn. The alpha diversity indexes were calculated. A dilution curve was constructed. We obtained the R-value by analyzing the distance matrix between samples. Finally, the composition and abundance of the gut microbiota were identified.

### Liquid Chromatography-Tandem Mass Spectrometry (LC-MS/MS)

Fecal samples from all subjects were centrifuged at 1,150 g and 4°C for 10 min. Then the supernatant was further divided into 100 μl, put into the labeled tube and stored at −80°C before preparing for metabonomics analysis. Each bisection sample was separated from 300 μl cold acetonitrile, mixed and then swirled for 30 s before analysis. The mixture was deproteinized by centrifugation at 4°C (21,130 g, 30 min), and 1 μl of supernatant was injected into UPLC. The ion source parameters and scanning parameters were optimized.

### Quantitative Real-Time PCR

About 0.02 g tissues in Trizol were put into a 1.5 ml centrifuge tube, and 1 ml of Trizol was added to the homogenizer to grind and homogenize thoroughly. About 500 μl of the cells in Trizol were placed in a 1.5 ml centrifuge tube. Fluorescence quantitative PCR (quantstudio1, Thermo, USA) was performed on a fluorescence quantitative PCR machine. The reaction conditions were pre-denaturation at 95°C for 10 min, denaturation at 94°C for 15 s, and annealing at 60°C for 30 s, a total of 40 cycles. The internal reference primer was β-actin, and the primer sequence was shown in Table 2.

**Table 2:** Primer sequences.

**Gene**	**Genbank number**	**Sequences (5^**′**^–3^**′**^)**
E-cadherin	NG_008021	F: ATTTTTCCCTCGACACCCGAT
		R: TCCCAGGCGTAGACCAAGA
N-cadherin	NC_000018	F: GGGAAATGGAAACTTGATGGCA
		R: TGGAAAGCTTCTCACGGCAT
Vimentin	NG_012413	F: CCCTTGACATTGAGATTGCCACC
		R: ACCGTCTTAATCAGAAGTGTCCT
AhR	NC_000007	F: CAACAGCAACAGTCCTTGGC
		R: GTTGCTGTGGCTCCACTACT
β-actin	NG_007992	F: ACCCTGAAGTACCCCATCGAG
		R: AGCACAGCCTGGATAGCAAC

### Western Blot

We cut 0.025 g tissues, and then washed the tissues with ice-cold PBS. We added 300 μl RIPA lysate and ground the tissues repeatedly in the biological sample homogenizer until no tissue masses were visible. For primary antibodies, we used rabbit anti-AhR (1:1,000, #83200, CST), rabbit anti-E-cadherin (1:5,000, 20874-1-AP, Proteintech), mouse anti-N-cadherin (1:6,000, 66219-1-Ig, Proteintech), rabbit anti-Vimentin (1:5,000, ab92547, Abcam), rabbit anti-PCNA (1:5,000, 10205-2-AP, Proteintech) and mouse anti-β-actin (1:5,000, 60008-1-Ig, Proteintech). We rinsed the membrane three times with TBST for 10 min each time. Then we incubated secondary antibodies HRP-conjugated Affinipure Goat Anti-Mouse IgG (H + L) (1:5,000, SA00001-1, Proteintech) and HRP-conjugated Affinipure Goat Anti-Rabbit IgG (H+L) (1:6,000, SA00001-2, Proteintech). The film was immersed in SuperECL Plus (K-12045-D50, Advansta, USA) for luminescence development.

### Cell Counting Kit-8 Assay

We used the CCK-8 kit (NU679, Dojindo Molecular Technologies, Inc., Japan) to analyze the cell viability. The cells were taken in a logarithmic growth phase and were digested with trypsin. At the density of 5 × 10^3^ cells/well, the cells were inoculated into a 96-well plate with 100 μl/well. Ten microliter CCK8 solution of complete culture medium was co-cultured with cells in each well. The absorbance (OD) value at 450 nm was analyzed by a Bio-Tek microplate reader after incubation with 5 % CO_2_ at 37°C for 4 h.

### Transwell Assay

We diluted Matrigel with 100 μl cold, serum-free DMEM medium per well. Then we placed 500 μl complete medium containing 10% FBS in the lower chamber. The cells were digested with trypsin to form a single cell. We put the upper chamber into a new hole with PBS. We used 0.1% crystal violet for 5 min.

### Flow Cytometry

Cells were collected with trypsin digestion solution (c0201, Beyotime, China) without EDTA. We collected about 2 × 10^5^ cells. Five hundred microliter of binding buffer was added to the cell suspension. We added 5 μl annexin V-FITC (kga108, keygen, China) and mixed well. Finally, we added 5 μl Propidium Iodide (PI) (mb2920, Meilunbio, China) into the mixture and mixed them well. Flow cytometry was used to observe the changes within 1 h.

### Immunofluorescence

The slices were cleaned 2~3 times with PBS. Then the slices were fixed with 4% paraformaldehyde for 30 min. The primary antibody AhR was dripped onto the slices at 4°C overnight. The second antibody was incubated by dropping 50–100 μl anti-rabbit IgG labeled fluorescent antibody. Then the slices were incubated at 37°C for 90 min. Finally, the slices were washed with PBS 3 times, 5 min each. DAPI solution was used to stain the nucleus at 37°C for 10 min. Then PBS was washed 3 times, 5 min each. The slices were placed under a fluorescent microscope for observation.

### Cell Cycle Assay

We took out the fixed sample. One microliter of pre-cooled PBS was added to the sample for cell suspension. We added 150 μl PI working solution into the cell solution and stained it at 4°C for 30 min. PI was excited by 488 nm argon ion laser and received by 630 nm pass filter. 1 × 10^4^ cells were collected by FSC/SSC scatterplot. Adhesion cells and fragments were excluded by gating technique. The percentage of cell cycle on PI fluorescence histogram was analyzed.

### Statistical Analysis

All experimental data were analyzed by GraphPad 8.0 software. Measurement data were expressed as mean ± standard deviation. The unpaired *T*-test was used between the two groups conforming to the normal distribution. One-way analysis of variance (ANOVA) was used for comparison between multiple groups. *P* < 0.05 was considered statistically significant.

## Discussion

This study found that RCC has a certain internal relationship with gut microbiota and tryptophan metabolite. Through cell experiments, we found that Kyn may promote EMT by activating AhR, further promote RCC cells migration and invasion and inhibit RCC cells apoptosis.

The gut microbiota is closely related to cancer (18). We found that the composition of gut microbiota in RCC patients was changed significantly. *Bacteroides* and *Akkermansia* were increased significantly, while *Blautia, Bifidobacterium*, and *Megamonas* were decreased significantly. Recent studies have shown that gut microbiota composition affects the success of immune checkpoint blocking therapy for RCC (19). Clinical studies have shown that patients treated with vascular endothelial growth factor tyrosine kinase inhibitor (VEGF-TKIs) combined with mRCC have higher *Bacteroides* and lower *Prevotella* (20). These results indicated that the gut microbiota composition changes were related to the occurrence and development of RCC.

Tryptophan catabolism has become a critical metabolic regulation factor for tumor progression (21). Different cancers prognosis showed that an essential indicator of tryptophan metabolism is serum KTR. When the serum KTR increased, tryptophan was metabolizing through indoleamine 2,3-dioxygenase 1 (IDO1) or tryptophan 2,3 -Dioxygenase (TDO) through the Kyn pathway. At the same time, other researchers have reported higher KTR in the serum of patients with advanced RCC or resistance to immune checkpoint inhibitor (22, 23). The composition of the gut microbiota determines several tryptophan metabolites because they are catabolism products of gut microbiota. These tryptophan-derived microbial catabolites are important signaling molecules in the host and the microorganisms (24, 25). Targeted metabolomics studies have shown that RCC patients have elevated Kyn pathway metabolites (26). Our experiment revealed that the distribution of tryptophan metabolites in gut microbiota changed significantly in RCC. Through correlation analysis, it was found that the tryptophan metabolites were correlated considerably with *Agathobacter, Escherichia-Shigella, Romboutsia*, and *Akkermansia*, etc. Studies have shown that microorganisms mediate gut Trp metabolism changes and participate in the occurrence of cancer (27). This was consistent with our research. Through correlation analysis, we also found that tryptophan metabolites of gut microbiota were significantly correlated with AhR and E-cadherin expressions in RCC. These results indicated that the Kyn metabolic pathway of the gut microbiota might be involved in the pathogenetic progress of RCC. This experiment assessed that the Kyn pathway was operable and could be used as a therapeutic target for RCC.

Kyn is the main product of the tryptophan metabolic pathway catalyzed by TDO2 and IDO in tumor cells. Kyn proved that AhR could be activated. AhR in an autocrine/paracrine manner can inhibit the anti-tumor immune response and promote tumor cell survival and movement (28). AhR was identified as a ligand-activated transcription factor of the basic helix-loop-helix (bHLH) Per-Arnt-Sim (PAS) family, and it played an important role in a wide range of physiological and pathological conditions (29–31). AhR participates in the induction of Slug expression, and this process inhibits E-cadherin expression. MMPs expression is also a target of the AhR pathway. Odibenzo-p-dioxin (TCDD) exposure up-regulated the expression and activity of MMP9 in a variety of malignant tumors, including melanoma cells, urothelial cancer cells, prostate cancer cells, and gastric cancer cells (32). AhR is involved in the induction of EMT by PCBs in HCC cells (33). In this study, we aimed to study the influence of AhR on the progress of EMT in RCC. Our results showed that AhR was highly expressed in RCC. In addition, Kyn could promote 786-O cells migration and invasion and inhibit 786-O cells apoptosis by activating AhR.

Fecal microorganisms and metabolites are often affected by diet (34). In this study, the subjects were not given a standardized diet like other studies (3) but kept the original eating habits, which is the limitation of this study. However, non-invasive research can be overcome by expanding the sample size. In addition, the effect of tryptophan metabolites of gut microbiota on the AhR activation pathway of the host itself was complex, which may require more evidence to prove its specific regulatory mechanism. Based on these factors, we will expand the sample size in future research and explore the influence of tryptophan metabolites of gut microbiota on RCC in combination with clinical and animal experiments.

In conclusion, through the research of this subject, we have verified that the RCC gut microbiota metabolism is disordered, and the Kyn metabolism is increased. *In vitro* experiments further confirmed that Kyn could promote 786-O cells migration and invasion and the progress of EMT and inhibit 786-O cells apoptosis by activating AhR. Our design clarified that the gut microbiota could activate AhR through its tryptophan metabolism to mediate the metastasis of RCC.

## Data Availability Statement

The original contributions presented in the study are publicly available. This data can be found here: https://www.ncbi.nlm.nih.gov/sra/PRJNA735071.

## Ethics Statement

All experiments were performed according to the guidelines set by the Medical Ethics Committee of Xiangya Hospital of Central South University (202105187), Changsha, Hunan, China. Written informed consent was obtained from all participates. The patients/participants provided their written informed consent to participate in this study.

## Author Contributions

GD, XC, and YH designed the study, performed the research, analyzed data, and wrote the paper. All authors contributed to the article and approved the submitted version.

## Conflict of Interest

The authors declare that the research was conducted in the absence of any commercial or financial relationships that could be construed as a potential conflict of interest.

## Publisher's Note

All claims expressed in this article are solely those of the authors and do not necessarily represent those of their affiliated organizations, or those of the publisher, the editors and the reviewers. Any product that may be evaluated in this article, or claim that may be made by its manufacturer, is not guaranteed or endorsed by the publisher.
